# Supplementary material for: Downregulation of Circulating Hsa-miR-200c-3p Correlates with Dyslipidemia in Patients with Stable Coronary Artery Disease
Source: Int J Mol Sci. 2023 Jan 6;24(2):1112. doi: 10.3390/ijms24021112 (PMC9865013; doi:10.3390/ijms24021112)
Supplement: Supplementary file 1 [file ijms-24-01112-s001.zip › ijms-1968491-supplementary.pdf]

Supplementary Table S1. List of the 50 Regulated miR (Fold-Change ≥ 1.5 and P-Value ≤ 0.05).

miR ID	Regulation	Fold Change	P-Value
hsa-miR-1246	up	9,30	5,18E-04
hsa-miR-374b-3p	up	5,86	1,62E-03
hsa-miR-3613-5p	up	5,42	4,29E-05
hsa-miR-22-3p	up	4,83	3,40E-04
hsa-miR-3940-3p	up	4,53	5,90E-03
hsa-miR-106a-3p	up	4,46	1,39E-02
hsa-miR-548h-3p	up	3,75	1,99E-02
hsa-miR-548z	up	3,75	1,99E-02
hsa-miR-4732-3p	up	3,37	1,64E-02
hsa-miR-4433b-5p	up	3,06	1,75E-02
hsa-miR-4685-3p	up	2,90	4,69E-02
hsa-miR-7976	up	2,88	4,12E-02
hsa-miR-1-3p	up	2,67	1,73E-02
hsa-miR-374a-3p	up	2,45	1,65E-02
hsa-miR-224-5p	up	2,26	2,65E-02
hsa-miR-339-5p	up	2,15	4,08E-02
hsa-miR-625-3p	up	2,12	3,29E-02
hsa-miR-625-5p	up	2,12	3,78E-02
hsa-miR-1185-1-3p	down	-22,45	2,13E-05
hsa-miR-4676-3p	down	-11,42	3,10E-03
hsa-miR-381-3p	down	-8,01	4,44E-04
hsa-miR-654-3p	down	-7,89	8,43E-05
hsa-miR-337-5p	down	-7,69	4,86E-04
hsa-miR-27a-5p	down	-6,22	2,97E-04
hsa-miR-410-3p	down	-5,59	3,89E-03
hsa-miR-376c-3p	down	-5,53	1,02E-03
hsa-miR-411-3p	down	-5,15	1,23E-03
hsa-miR-323a-3p	down	-4,95	4,45E-03
hsa-miR-4646-5p	down	-4,93	1,16E-03
hsa-miR-29a-5p	down	-4,39	7,45E-03
hsa-miR-589-3p	down	-3,94	1,07E-02
hsa-miR-338-5p	down	-3,92	2,18E-03
hsa-miR-9-5p	down	-3,90	1,53E-02

hsa-miR-376a-3p	down	-3,89	8,80E-03
hsa-miR-329-3p	down	-3,80	9,44E-03
hsa-miR-1287-3p	down	-3,62	3,24E-02
hsa-miR-655-3p	down	-3,60	1,56E-02
hsa-miR-1273a	down	-3,26	8,64E-03
hsa-miR-337-3p	down	-3,15	3,55E-02
hsa-miR-539-5p	down	-3,13	2,27E-02
hsa-miR-181a-3p	down	-3,13	3,37E-02
hsa-miR-200c-3p	down	-2,97	9,11E-03
hsa-miR-4485-5p	down	-2,87	4,98E-02
hsa-miR-411-5p	down	-2,75	3,02E-02
hsa-miR-491-5p	down	-2,50	3,95E-02
hsa-miR-335-3p	down	-2,45	3,82E-02
hsa-miR-3653-3p	down	-2,44	1,56E-02
hsa-miR-574-5p	down	-2,24	1,78E-02
hsa-miR-150-3p	down	-2,21	4,31E-02
hsa-miR-1260a	down	-2,07	4,88E-02
